# Cell-to-Cell Heterogeneity in Cortical Tension Specifies Curvature of Contact Surfaces in *Caenorhabditis elegans* Embryos

**DOI:** 10.1371/journal.pone.0030224

**Published:** 2012-01-10

**Authors:** Masashi Fujita, Shuichi Onami

**Affiliations:** 1 Laboratory for Developmental Dynamics, RIKEN Quantitative Biology Center, Kobe, Japan; 2 Developmental Systems Modeling Team, RIKEN Advanced Science Institute, Yokohama, Japan; Georgia State University, United States of America

## Abstract

In the two-cell stage embryos of *Caenorhabditis elegans*, the contact surface of the two blastomeres forms a curve that bulges from the AB blastomere to the P_1_ blastomere. This curve is a consequence of the high intracellular hydrostatic pressure of AB compared with that of P_1_. However, the higher pressure in AB is intriguing because AB has a larger volume than P_1_. In soap bubbles, which are a widely used model of cell shape, a larger bubble has lower pressure than a smaller bubble. Here, we reveal that the higher pressure in AB is mediated by its higher cortical tension. The cell fusion experiments confirmed that the curvature of the contact surface is related to the pressure difference between the cells. Chemical and genetic interferences showed that the pressure difference is mediated by actomyosin. Fluorescence imaging indicated that non-muscle myosin is enriched in the AB cortex. The cell killing experiments provided evidence that AB but not P_1_ is responsible for the pressure difference. Computer simulation clarified that the cell-to-cell heterogeneity of cortical tensions is indispensable for explaining the pressure difference. This study demonstrates that heterogeneity in surface tension results in significant deviations of cell behavior compared to simple soap bubble models, and thus must be taken into consideration in understanding cell shape within embryos.

## Introduction

The cell-cell contact surfaces in developing embryos are often curved like arcs. Those of the two-cell stage embryos of *C. elegans* exhibit this pattern. The embryos at this stage have a larger anterior blastomere (AB) and a smaller posterior blastomere (P_1_) [1]. The contact surface is flat in the early two-cell stage but gradually curves in the late two-cell stage, forming a bulge from AB toward P_1_ [2].

Soap bubbles have long been recognized as a model for explaining the shapes of biological cells [3]. Various systems of biological cells adopt a geometry similar to that of soap bubbles [4]–[7]. Curved surfaces are also common in soap bubbles, and their curvature is explained by the internal pressures of bubbles as follows [8]. The internal pressures differ from bubble to bubble, and thus, two neighboring bubbles have different internal pressures. The bubble with higher internal pressure pushes its boundary surface toward the bubble with lower internal pressure, generating the curvature of the boundary surface.

The above pressure-based explanation also applies to the surface curvature of the two-cell stage embryo of *C. elegans*. A previous study reported that when the two cells were artificially fused by laser irradiation, a flow of cytoplasm from AB into P_1_ was observed [9]. This direction of the flow indicates that the intracellular hydrostatic pressure of AB is higher than that of P_1_. Therefore, the intracellular hydrostatic pressures of the cells successfully explain the bulging of the curved contact surface from AB to P_1_.

However, from the viewpoint of the soap bubble model, it is puzzling that AB has higher pressure than P_1_. The soap-bubble analogy of the two-cell stage embryo is two bubbles in contact (“double bubble”). In the double bubble, the volumes of the bubbles determine the entire geometry, including the shape of the boundary surface. The boundary surface curves from the smaller bubble toward the larger one because the smaller bubble has a higher internal pressure than the larger one. In contrast, the opposite holds true for embryos in which the larger cell (AB) has higher pressure than the smaller cell (P_1_). The reason for this contradiction is unclear. By studying this contradiction, an important difference between soap bubbles and biological cells might be clarified.

In this study, we investigated the mechanism by which AB acquires higher pressure than P_1_. Chemical and genetic interferences demonstrated that the pressure difference is mediated by actomyosin. Fluorescence imaging and laser ablation data suggested that the AB-specific increase in cortical tension underlies the pressure difference. Computer simulation confirmed that the higher tension of AB is sufficient to explain the curved contact surface and the pressure difference. Our study demonstrated that the cell-to-cell heterogeneity of cortical tension is a nonnegligible difference between bubbles and cells for understanding cell shape within embryos.

## Results

### Contact surface curves in the late two-cell stage

**Figure 1:**
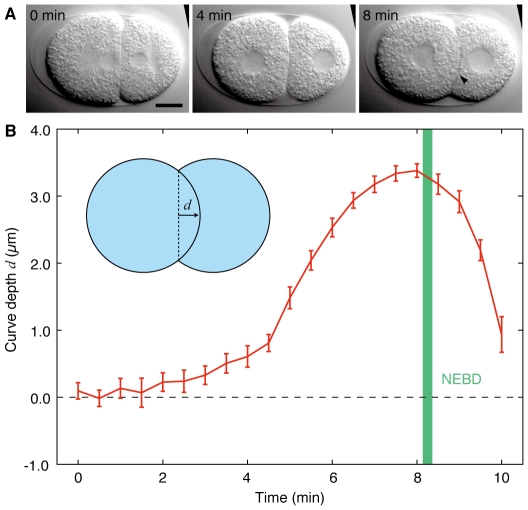
Contact surface curves into the posterior blastomere. (A) Images from time-lapse DIC microscopy of a wild-type N2 embryo. The anterior end is oriented to the left. The larger anterior cell is AB, and the smaller posterior cell is P_1_. Arrowhead indicates a curved contact surface. (B) Depth of the curved contact surface *d* as a function of time during the two-cell stage. Inset shows the definition of *d*. Green, mean±s.e.m. interval of NEBD of AB. Error bars indicate s.e.m. n = 20. Time is from the onset of the two-cell stage. Scale bars, 10 µm.

We observed the shapes of contact surfaces in the two-cell stage wild-type embryos (Figure 1A). When cytokinesis in the one-cell stage was complete, the contact surface between AB and P_1_ was flat (Figure 1A, left). The contact surface then gradually curved, forming a bulge from AB to P_1_ (Figure 1A, right). After that, the contact surface became flat again and AB initiated cytokinesis. We measured the curve depth of the contact surfaces at 30-s intervals (Figure 1B, n = 20). Eight minutes after the onset of the two-cell stage, the curve reached its maximum depth of 3.4±0.1 µm (mean±s.e.m.; ±denotes s.e.m. throughout). We also observed that NEBD of AB occurred 8.2±0.1 min after the onset of the two-cell stage.

### Curvature of the contact surface is related to the intercellular pressure difference

We examined whether the shape of the contact surface depended on the pressure difference between the cells. The magnitude of intercellular pressure difference will be reflected in the cytoplasmic flow associated with laser-induced cell fusion. We performed the fusion experiments either before or after curve formation.

**Figure 2:**
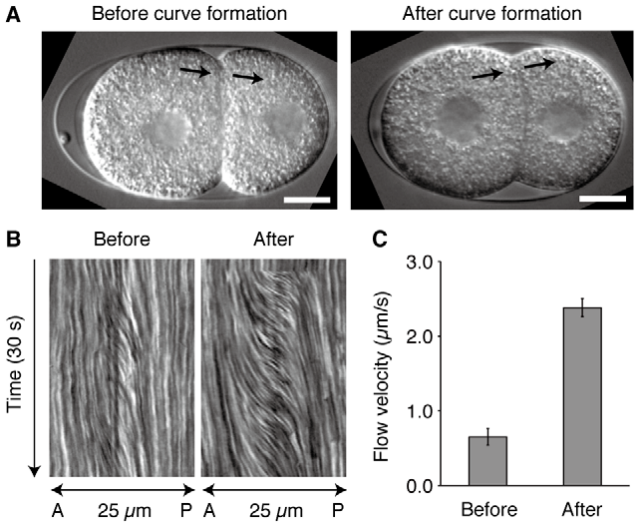
Laser-induced cell fusion is accompanied by cytoplasmic flow. (A) AB and P_1_ were laser fused either before (left) or after (right) their contact surfaces formed curves. Arrows indicate the location and direction of the observed flow of cytoplasmic granules. Scale bars, 10 µm. (B) Kymographs of cytoplasmic flow. A, anterior; P, posterior. (C) The velocity of cytoplasmic flow before and after curve formation (n = 10 each). Error bars indicate s.e.m.

In either case, cytoplasmic flow was observed after laser irradiation and the flow direction was from AB to P_1_, which is consistent with previous reports [9] (Figure 2A, Video S1 and S2). However, the flow of cytoplasmic granules after curve formation was much faster than that before curve formation. We generated the kymograph of the flows (Figure 2B) and quantified the flow velocity from the kymograph (Figure 2C). The result confirmed that the flow velocity after curve formation (2.4±0.1 µm/s, n = 10) was significantly faster than that before curve formation (0.6±0.1 µm/s, n = 10; *P*<0.0001, Mann–Whitney test). This indicates that the pressure difference across curved contact surfaces is larger than that across flat contact surfaces. These results demonstrate that the curvature of the contact surface is generated by the intercellular pressure difference.

### Intercellular pressure difference is dependent on actin filaments

**Figure 3:**
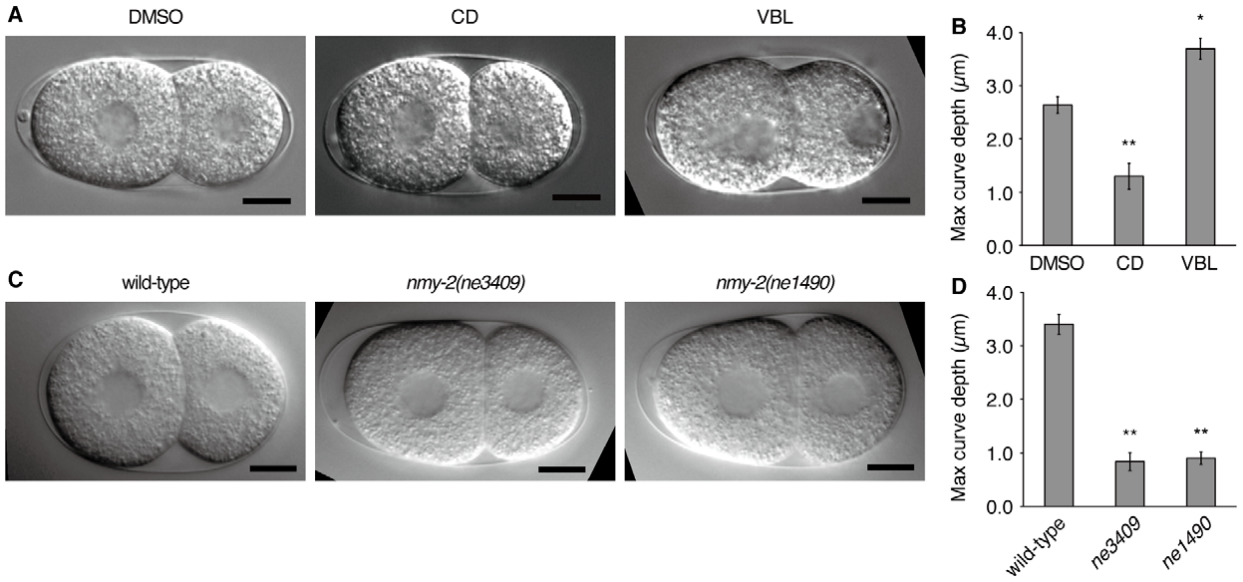
Curvature of the contact surface is dependent on actomyosin. (A) DIC images of embryos treated with cytoskeletal inhibitors. CD, cytochalasin D; VBL, vinblastine. Images were taken 8 min after the onset of the two-cell stage. (B) Maximum curve depth of the drug-treated embryos (n = 10 each). (C) DIC images of ts mutants of *nmy-2* at the restrictive temperature. Images were taken immediately before NEBD of AB. (D) Maximum curve depth in the *nmy-2* embryos at the restrictive temperature (n = 10 each). Error bars indicate s.e.m. *, *P*<0.01; **, *P*<0.001; Mann–Whitney test. Scale bars, 10 µm.

To investigate the molecular mechanism underlying the intercellular pressure difference, we examined the effects of cytoskeletal inhibitors on curve formation. Embryos were permeabilized in a drug-containing medium at the early two-cell stage, and the shape of the contact surfaces was observed (Figure 3A). In the late two-cell stage, embryos treated with vehicle (DMSO) had curved contact surfaces that were apparently normal (n = 10). Inhibition of microtubules with vinblastine (VBL) had no apparent effect on the curved contact surfaces (n = 10). However, disruption of actin filaments by cytochalasin D (CD) significantly impaired curve formation between the contact surfaces (n = 10).

We quantified the depth of the curve in these embryos and compared the maximum depth until NEBD of AB (Figure 3B). The curve depth of vehicle-treated embryos was 2.6±0.2 µm, which was smaller than that of untreated embryos, presumably because of DMSO or artificial medium. The curve depth of VBL-treated embryos was 3.7±0.2 µm, which was larger than that of vehicle-treated embryos (*P*<0.01, Mann–Whitney test). The curve depth of CD-treated embryos was 1.3±0.2 µm, which was significantly smaller than that of vehicle-treated embryos (*P*<0.001). These results suggest that although microtubules might have some regulatory roles, actin filaments are essential for the pressure difference across curved contact surfaces.

### Intercellular pressure difference is dependent on non-muscle myosin

Mechanical processes involving actin filaments are often associated with force generation by non-muscle myosin [10]. To examine the role of non-muscle myosin in the difference in intercellular pressure between AB and P_1_, two temperature-sensitive (ts) mutants of non-muscle myosin, *nmy-2(ne3409)* and *nmy-2(ne1490)* [11], were studied. The ts embryos were maintained at the permissive temperature until the onset of the two-cell stage. Subsequently, the embryos were transferred to the restrictive temperature and observed by DIC microscopy. The contact surfaces of the ts mutants were less curved throughout the two-cell stage compared with those of the wild-type embryos (Figure 3C). Quantification of the curve depth confirmed this trend (*ne3409*: 0.8±0.2 µm; *ne1490*: 0.9±0.1 µm; wild type: 3.4±0.2 µm; Figure 3D). Therefore, the intercellular pressure difference is dependent on non-muscle myosin.

### Non-muscle myosin exhibits cortical localization that increases specifically in AB

**Figure 4:**
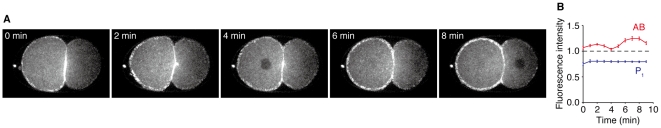
Dynamics of NMY-2::GFP in the two-cell stage embryos. (A) Z-stack projections from a time-lapse sequence of an embryo expressing NMY-2::GFP. The maximum intensity projection of four planes spanning 1.5 µm is shown. (B) Mean fluorescence intensity of NMY-2::GFP in AB and P_1_ (n = 12). Values normalized by the spatiotemporal average of both cells are shown. Error bars indicate s.e.m. Time is from the onset of the two-cell stage.

To investigate how myosin mediates the intercellular pressure difference, we imaged the localization of non-muscle myosin using GFP-tagged NMY-2 (NMY-2::GFP) [12] and spinning disk confocal microscopy. NMY-2::GFP accumulated at the cell cortex throughout the two-cell stage, and the cortex of AB was brighter than that of P_1_ (Figure 4A, n = 12). This cortical localization suggests that NMY-2 contributes to cortical tension.

Additionally, we observed that GFP fluorescence at the AB cortex increased in the late two-cell stage (Figure 4A). To confirm this, we quantified the fluorescence intensity in each cell from the maximal intensity projections of the confocal images (Figure 4B). The results revealed that the mean intensity in AB at the late two-cell stage (1.25±0.03 at 8 min) was significantly higher than that at the early two-cell stage (1.13±0.02 at 2 min; *P*<0.05, Wilcoxon signed rank test). In contrast, the mean intensity in P_1_ (0.80±0.02 at 2 min) was almost constant and remained lower than that in AB (*P*<0.001 at 2 min). The observed dynamics of NMY-2 suggest that the cortical tension of AB increases in the late two-cell stage, whereas that of P_1_ changes minimally.

### Cell killing indicates that AB but not P_1_ is responsible for the intercellular pressure difference

**Figure 5:**
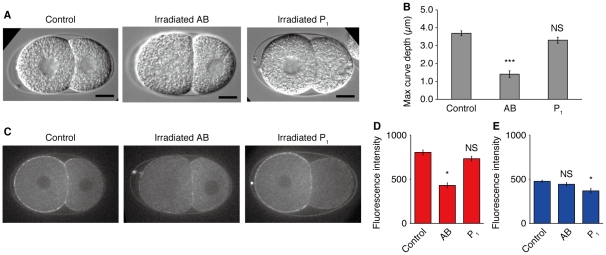
UV irradiation to the AB nucleus impairs curve formation. (A) DIC images of the embryos in which their nuclei were irradiated by a UV laser. The images were taken 8 min after the onset of the two-cell stage. Scale bars, 10 µm. (B) Maximum curve depth of the contact surfaces. AB, UV irradiation to the AB nucleus (n = 10); P_1_, UV irradiation to the P_1_ nucleus (n = 10). (C) Embryos that expressed NMY-2::GFP and their nuclei were irradiated by UV. 8 min after the onset of the two-cell stage. (D and E) Fluorescence intensity at the cell cortex. Control, untreated embryos (n = 6); AB, UV irradiation to the AB nucleus (n = 7); P_1_, UV irradiation to the P_1_ nucleus (n = 5). (D) Cortex of AB cell. (E), Cortex of P_1_ cell. Error bars indicate s.e.m. *, *P*<0.01; ***, *P*<0.0001; NS, non-significant (*P*>0.1); Mann–Whitney test.

The above results suggest that the time-dependent change in AB but not in P_1_ is essential for the pressure difference. If this is the case, curve formation would be impaired when the function of AB is disturbed. This hypothesis was tested by selectively killing cells by laser ablation. The nucleus of each cell in the early two-cell stage was irradiated with the UV laser, and then curve formation was monitored (Figure 5A). As expected, irradiated AB embryos did not form curved contact surfaces (n = 10), whereas irradiated P_1_ embryos formed apparently normal curved surfaces (n = 10). The curve depth was quantified (Figure 5B), and it was confirmed that irradiated AB embryos have significantly smaller depths (1.4±0.2 µm) than untreated embryos (3.7±0.1 µm, n = 10; *P*<0.0001, Mann–Whitney test). No significant difference was observed in curve depth between irradiated P_1_ embryos (3.3±0.2 µm) and untreated embryos (*P* = 0.11). This result indicates that the pressure difference is generated by AB and not P_1_.

We examined how irradiation with UV affected the localization of NMY-2::GFP. We found that NMY-2::GFP still localized to cortex even in irradiated cells but that the cortical fluorescence of irradiated AB cells were noticeably reduced (Figure 5C). To confirm this, the fluorescence intensities at the cell cortex were quantified (Figure 5D and E). Regarding the fluorescence intensity at AB cortex, control embryos had values of 804±27, whereas the irradiated AB embryos had significantly smaller values of 430±29 (*P*<0.01; Figure 5D). Similarly, the fluorescence intensities of the P_1_ cortex were 477±12 for control embryos and 369±28 for the irradiated P_1_ embryos, having a significant difference (*P*<0.01; Figure 5E). In contrast, the AB cortex of irradiated P_1_ embryos (733±28) and the P_1_ cortex of irradiated AB embryos (443±20) did not have significant differences with the control (*P* = 0.13 and *P* = 0.25, respectively). These results suggest that myosin activity in AB is responsible for the intercellular pressure difference.

### Computer simulation of the cell shapes and the intracellular pressures

The results obtained so far suggests that cortical tensions are different between AB and P_1_, and this heterogeneity of cortical tensions plays an essential role in the pressure difference between the cells. In order to investigate whether this heterogeneity can explain the pressure difference, we performed the computer simulation of the cell shapes and the intracellular pressures.

**Figure 6:**
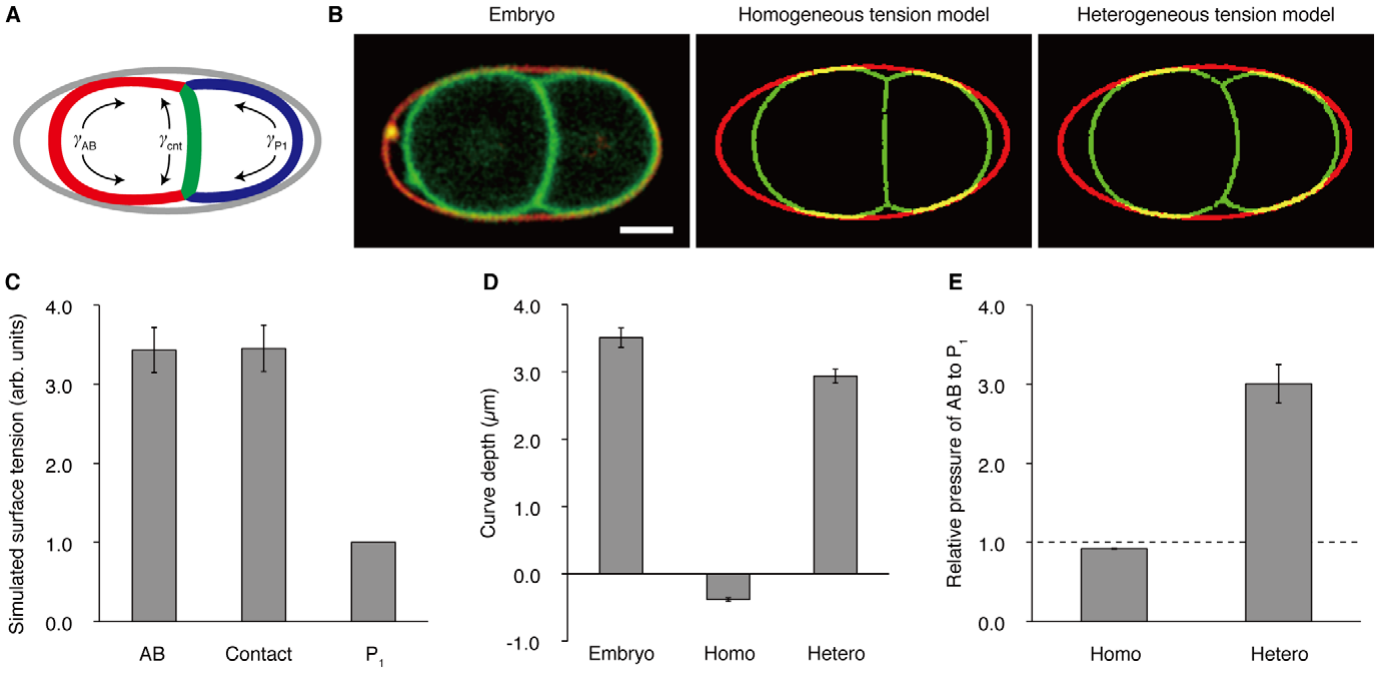
Computer simulation of the homogeneous and heterogeneous tension models. (A) Graphical representation of the simulation model. Gray, the eggshell; red, the outer surface of AB; blue, the outer surface of P_1_; green, the contact surface. *γ*_AB_, *γ*_P1_, and *γ*_cnt_ denote the surface tension parameters. (B) Left, a confocal image of an embryo that has the GFP-tagged plasma membranes and the TRITC-stained eggshell. Center, a simulated cell shape using the homogeneous tension model. Right, a simulated cell shape using the heterogeneous tension model. (C) Surface tension parameters that made the heterogeneous tension model most similar to the GFP::PH embryos. The results of 20 simulations that correspond to the 20 observed embryos are shown. AB, surface tension parameter *γ*_AB_. Contact, surface tension parameter *γ*_cnt_. P_1_, surface tension parameter *γ*_P1_. (D) Curve depth of the contact surfaces. Embryo, GFP::PH embryos; Homo, the homogeneous tension model; Hetero, the heterogeneous tension model. (E) Ratios of the simulated pressures between AB and P_1_. The values indicated are the ratio *P*_AB_/*P*_P1_, where *P*_AB_ is the pressure inside AB, *P*_P1_ is the pressure inside P_1_. Error bars indicate s.e.m. Scale bars, 10 µm.



We modeled the two-cell stage embryos as a system of surfaces confined to an ellipsoid (Figure 6A). The surfaces of our model consisted of three parts: the outer (contact-free) surface of AB, the outer surface of P_1_, and the contact surface. We also assumed that the cells adopt the shapes in which their mechanical energies are minimized, as has been reported for other cells [13]–[15]. We defined the mechanical energy of this system as

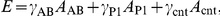



where *E* is the total energy, *γ*_AB_, *γ*_P1_ and *γ*_cnt_ are the surface tension parameters, and *A*_AB_, *A*_P1_ and *A*_cnt_ are the surface area of the outer AB, outer P_1_ and contact surfaces, respectively. This model can be classified into two subclasses depending on the parameter choice. The first one is the “homogeneous tension model”, in which the three surfaces have the same magnitude of surface tensions (i.e. *γ*_AB_ = *γ*_P1_ = *γ*_cnt_). The second one is the “heterogeneous tension model”, in which the three parameters *γ*_AB_, *γ*_P1_ and *γ*_cnt_ can adopt independent values.

First, we examined which of the two models, homogeneous or heterogeneous tension models, has the ability to reproduce the cell shape in embryos (Figure 6B). We acquired the three-dimensional cell shapes of GFP::PH embryos [16] (n = 20), and performed computer simulations under the constraints of the observed cell volumes and eggshells. The homogeneous tension model failed to reproduce the contact surface that bulges from AB to P_1_. Rather, its contact surface was slightly curved to the opposite direction. In contrast, the heterogeneous tension model was able to reproduce the overall cell shape including the curved contact surface, when the surface tension parameters were set to appropriate values. The parameter values that reproduced the cell shapes most accurately were *γ*_AB_ = 3.4±0.3 and *γ*_cnt_ = 3.4±0.3 as relative values to *γ*_P1_ (Figure 6C).

We quantified the curve depths in the GFP::PH embryos and those in the cell shapes simulated by the two models (Figure 6D). The curve depths in the embryos were 3.5±0.1 µm. In the homogeneous tension model, the curve depths were −0.4±0.03 µm. Its negative value confirmed that the contact surfaces simulated using the homogeneous tension model were curved from P_1_ to AB. The curve depths simulated by the heterogeneous tension model were 2.9±0.1 µm.

Next, we compared the intracellular pressures predicted by the two models (Figure 6E). In the homogeneous tension model, the pressure ratio between AB and P_1_ was 0.9±0.005, predicting a slightly higher pressure inside P_1_. In the heterogeneous tension model, the ratio was 3.0±0.2 and consistent with the higher pressure inside AB observed using the laser-induced cell fusion (Figure 2C). Taken together, the heterogeneous tension model was sufficient to explain the curve depth and the pressure difference, which the homogeneous tension model failed to explain.

### Cell size manipulation shows that the asymmetry in cell contents is important for the intercellular pressure difference

**Figure 7:**
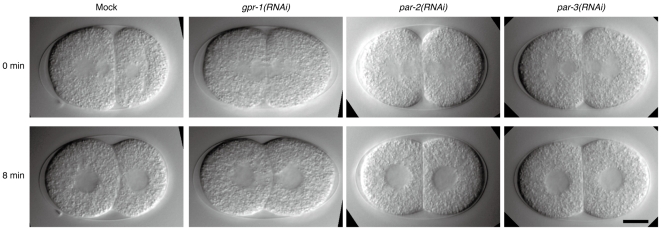
Cell size manipulation confirmed that the ratio of cell size is not the determinant of the pressure difference. DIC images of RNAi embryos. Time is from the onset of the two-cell stage. Cell size symmetry was judged at 0 min. Scale bars, 10 µm.

The first cleavage of *C. elegans* embryos is asymmetric, producing two daughters that are different both in size and contents. To assess the possible contribution of cell size ratio to the intercellular pressure difference, we generated embryos whose two-cell stage had symmetrical cell size. Such embryos could be produced by interfering asymmetric division at the one-cell stage, and depending on interfered genes, the contents of the cells would also become either symmetric or asymmetric. The genes *par-2* and *par-3* are central to the asymmetric division [17], [18], and their knockdown will lead to symmetric daughters not only in cell size but also in cell contents [19]. In contrast, *gpr-1* is an important gene for asymmetric spindle positioning but works downstream of the PAR proteins [20]–[22]. The knockdown of *gpr-1* equalizes cell size in the two-cell stage but would have less impact on the contents asymmetry than *par-2* and *par-3* [22]. We interfered these genes by RNAi and observed embryos with DIC optics (Figure 7). In mock embryos, 6 of 6 had asymmetrical cell size and also formed curved contact surfaces. In the case of *par-2(RNAi)* embryos, we found 7 embryos that had symmetrical cell size (among 10 embryos we observed), and 5 of 7 had flat contact surfaces. For *par-3(RNAi)*, we observed 7 embryos having symmetrical size (among 7 embryos we observed), and all of them had flat contact surfaces. In contrast, for *gpr-1(RNAi)*, we found 5 embryos having symmetrical size (out of 10 observed embryos), and 5 of 5 had curved contact surfaces. These results showed that cell size is not the determinant of pressure difference and suggested that contents asymmetry is indispensable to the pressure difference. Although we have not examined NMY-2 asymmetry in the RNAi embryos, the contents asymmetry might lead to cortical heterogeneity. The above observations are consistent with our view that the heterogeneity of cortical tension is important for explaining cell shape.

## Discussion

In soap bubbles, a curved boundary surface reflects the pressure difference across the surface, whereas a flat surface indicates no or little pressure difference. A previous study reported the occurrence of fusion-associated cytoplasmic flow in two-cell stage embryos [9], confirming the pressure difference between the cells. However, it was unclear whether the pressure difference was related to the surface curvature. We performed fusion experiments at two defined time points and observed small pressure differences when the contact surface was flat and large differences when the surface was curved (Figure 2C). These results confirmed that surface curvature is related to the pressure difference between the cells, similar to the mechanism in soap bubbles. Our next question was how the pressure difference is generated and why AB has higher pressure than P_1_ despite having a larger volume.

The intracellular hydrostatic pressure is often mediated by actomyosin [23], [24]. Our experiments of chemical inhibition of actin polymerization and genetic inhibition of NMY-2 revealed that the pressure difference in the late two-cell stage is mediated by actomyosin (Figure 3B and 3D). Interestingly, we observed that drug-induced disruption of microtubules increased the curve depth (Figure 3B). Microtubule inhibitors are known to increase the activity of the small GTPase Rho [25], [26], which enhances actomyosin contractility. The increase in curve depth upon microtubule disruption might be mediated by stimulation of the Rho pathway.

Actomyosin constitutes the cell cortex and generates a contractile force. This force, called cortical tension, plays a role analogous to the surface tension of soap bubbles [27]. Our fluorescence imaging data indicated that NMY-2::GFP localized to the cell cortex and the fluorescence intensity at the AB cortex increased in the late two-cell stage (Figure 4A). This result suggests that cortical tension increases specifically in AB in the late two-cell stage. This AB-specific change will be the cause of the higher pressure in AB. Cell-killing experiments also supported that AB but not P_1_ has an essential role in generating the pressure difference (Figure 5B).

In addition to actomyosin contractility, adhesion proteins can modulate cell surface tension [27], and it has previously been shown that the adhesion protein HMR-1/E-cadherin is portioned asymmetrically to the anterior half of the one-cell embryo [28], suggesting that AB might have higher levels of HMR-1 than P_1_. However, cadherin would be unrelated to our case because, in contrast to integrins that constitute cell-ECM adhesion, cadherin mediates cell-cell adhesion through homotypic binding. Therefore, in the two-cell stage, the only place where cadherin can work is the contact surface between AB and P_1_. This means that cadherin cannot explain the tension difference between the cells.

Our computer simulation showed that, if AB had about 3 times higher tension than P_1_ (Figure 6C), the computational model successfully reproduced the experimentally observed cell shapes and higher pressure of AB (Figure 6B and 6E). In contrast, when we assumed that all the surfaces had the same magnitudes of tensions, the model failed to reproduce the shapes and pressures of the cells. This indicates that the higher tension of AB is responsible for both the curved contact surface and the pressure difference between the cells. However, it should be also noted that the coincidence between the embryos and the heterogeneous tension models was not perfect, and there was a slight discrepancy in their curve depths (Figure 6D). This discrepancy might be due to the measurement errors of the cell volumes or the approximation of the eggshells as ellipsoids, but the exact reason is unclear.

In the case of soap bubbles, smaller bubble bulges into larger one, forming a curvature opposite to that in embryos. It raises a question about why such reversed curvature was not observed when actomyosin cortex was disrupted or AB was irradiated with UV. This apparent contradiction can be explained by smallness of the cell size effect on pressure difference. Indeed, our computer simulation predicted that the reversed curvature generated by homogeneous tensions would be quite small (Figure 6B). The disruption of cortex would produce a similar condition to the homogeneous tension model, and therefore large reversion was not observed. For AB killing, residual tension of AB would have a role. In the irradiated AB embryos, myosin still localized at the AB cortex (Figure 5C). Moreover, fluorescence intensity at the AB cortex (430±29) was comparable to that at the P_1_ cortex (443±20; *P* = 0.50, Wilcoxon signed rank test; Figure 5D and 5E). These data suggests that myosin activity at the AB cortex still remained even after the irradiation, and this residual tension would prevent the formation of reversed curvature.

**Figure 8:**
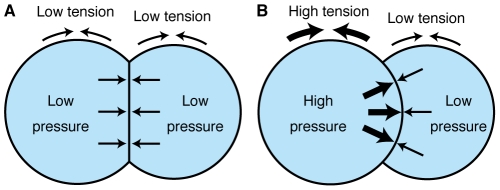
A model for the curved contact surfaces in the two-cell stage embryos. (A) Early two-cell stage. (B) Late two-cell stage.

Laplace's law dictates that the pressure difference across a fluid interface is proportional to the surface tension [8]. This implies that the intracellular hydrostatic pressure of AB will increase as the cortical tension of AB increases. On the basis of this idea, we propose a model for the pressure generation and surface curvature in the two-cell stage embryo. In the early two-cell stage, the magnitudes of cortical tension are approximately the same for AB and P_1_ (Figure 8A). Therefore, the intracellular hydrostatic pressures of both cells are also similar and the contact surface is flat. In the late two-cell stage, the cortical tension of AB increases, whereas that of P_1_ remains essentially unchanged (Figure 8B). The pressure in AB becomes higher than that in P_1_ and pushes the contact surface posteriorly, making the surface curved.

This study highlighted that one of the essential differences between biological cells and soap bubbles is heterogeneity among cells in terms of cortical tension. In soap bubbles, all surfaces have the same magnitude of surface tension. Even biological cells might have the same cortical tension when they consist of homogeneous cell types, as observed in some tissues. In such cases, the soap bubble model may sufficiently explain cell shape. In general, however, the mechanical properties of cell surfaces will differ from cell to cell. This is especially true for developing embryos, which contain heterogeneous cell types. To understand cell shapes in embryos, it will be important to consider such heterogeneity.

## Materials and Methods

### *C. elegans* strains, culture, and sample preparation

Strains were maintained according to standard procedures [29]. Temperature-sensitive strains were maintained at 15°C. All other strains were maintained at 22°C. The following strains and alleles were used in this study: Bristol N2, *zuIs45*[NMY-2::GFP], *nmy-2(ne3409ts)*, and *nmy-2(ne1490ts)*, *ltIs38*[GFP::PH(PLC1δ1)]. Embryos were mounted using an agar pad or poly-l-lysine. For agar pad mounting, embryos were transferred onto a 2% agarose pad and covered with a coverslip. For poly-l-lysine mounting, embryos were transferred onto a poly-l-lysine-coated coverslip and inverted over a microscope slide with four spots of high-vacuum grease (Dow Corning). In both types of mounting, the vacant space between the coverslip and slide was filled with M9 buffer (or another medium if indicated) and sealed with petroleum jelly.

### Measurement of curve depth

Wild-type embryos were prepared using the agar pad and mounted on an Olympus BX51 microscope equipped with an Olympus ×100 NA1.35 oil objective. DIC images were recorded using a Hamamatsu Orca CCD camera at 5-s intervals. The definition of the curve depth *d* is depicted in Figure 1B (inset). The depth is defined to be positive when the direction of the bulge is from AB to P1 and negative for the opposite direction. Depth measurements were performed at 30-s intervals using ImageJ.

### Laser ablation microscope

For cell fusion, drug treatments, and cell-killing experiments, the Leica LMD microscope equipped with an N_2_ laser (λ = 337 nm) and the Leica HCX APO ×100 NA1.30 oil objective was used. Time-lapse DIC images were acquired using the Leica DFC 360FX camera.

### Cell fusion and cell killing

In cell fusion experiments, wild-type embryos were prepared using the agar pad and mounted on the laser ablation microscope. The center plane of the embryo was imaged at 100-ms intervals using DIC optics. A UV laser was used to irradiate a peripheral site of the contact surface several times 4 or 8 min after the completion of P_0_ cytokinesis. A rectangular region elongated along the AP axis and centered at the irradiation site was clipped from the time-lapse images, and a kymograph was generated. The kymograph showed the path lines of yolk granules, and three path lines that exhibited steep changes were selected. The velocity was calculated from the gradient of each path line, and the mean of the three values was considered as the velocity of cytoplasmic flow.

For cell-killing experiments, embryos were mounted as described for cell fusion. The UV laser was used to irradiate the nucleus of AB or P_1_ before the onset of curve formation. The measurement of curve depth was initiated immediately after irradiation and continued at 30-s intervals until nuclear envelope breakdown (NEBD) of the other intact nucleus. Finally, the maximum value among the measured depths was extracted.

### Drug treatments

Embryos were mounted with poly-l-lysine and soaked in growth medium [30]. In the growth medium, we omitted fetal calf serum and added 1.5% stock salts (0.7 M NaCl, 0.3 M KCl) to adjust tonicity and 10 µM calcein-AM (Wako) to assess the success of perforation. This medium supported continuous cell divisions of permeabilized embryos for 2 h or longer. Cytochalasin D (Sigma) or vinblastine (Sigma) was diluted in DMSO and added at a final concentration of 5 or 10 µg/ml, respectively. The final concentration of DMSO in the medium was 1.1%.

The slide was placed under the laser ablation microscope and imaged using DIC optics. In the early two-cell stage, the eggshell and vitelline membrane were perforated by irradiation with the UV laser. Imaging was continued for 1 h, and if any cells were lysed, then data for those cells were discarded. After imaging, successful perforation was confirmed by calcein-AM fluorescence inside cells. The measurement of the curve depth was performed at 30-s intervals until NEBD of AB, and the maximum depth was obtained.

### Analysis of temperature-sensitive mutants

For sample preparation, a stereomicroscope equipped with a temperature-controlled plate (Tokai Hit, Japan) was used, and the plate was maintained at 15°C. An adult hermaphrodite was dissected, and embryos were mounted using the agar pad on the plate. The slide was maintained at 15°C until the onset of the two-cell stage and kept at this temperature for an extra 2 min to prevent regression of the cleavage furrow. The slide was then detached from the temperature-controlled plate and moved to the stage of the Olympus BX51 microscope at a room temperature (22–25°C). DIC imaging was performed using the Olympus ×100 NA1.35 oil objective and Hamamatsu Orca CCD camera. The curve depth of the contact surface was measured at 30-s intervals until NEBD of AB, and the maximum depth was obtained.

### Fluorescence imaging of myosin

Embryos expressing NMY-2::GFP were mounted using the agar pad. Then, 3D time-lapse images were acquired by a spinning disk confocal microscope (Olympus BX51 and Yokogawa CSU21) using the Olympus ×100 NA1.35 oil objective. Images were captured using an EMCCD camera (Andor DU897) at an EM gain of 300. The exposure time was 80 ms and laser power was 13 mW. Z-stacks at the cortex with 15 optical slices separated by 0.5 µm were collected at 1-min intervals using a piezo scanner (PI PIFOC). Hatching was confirmed on the next day.

To quantify fluorescence intensity, the maximal intensity projections of the Z-stacks were computed. For each projection image, a background region was selected and its mean intensity was subtracted from that of the whole image. Regions inside AB and P_1_ were manually clipped from the images and their mean fluorescence intensities *I*_AB_(*t*) and *I*_P1_(*t*) were calculated. These values contained large inter-sample variance. Our objective was to find a time-dependent behavior that was common to all samples, but the variance made the common behavior less visible. Therefore, the mean fluorescence intensity in the merged region, 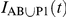, was computed and its time average, 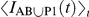, was used to normalize *I*_AB_(*t*) and *I*_P1_(*t*) in each sample.

Fluorescence imaging of myosin after cell killing was performed as follows. The laser ablation microscope was equipped with the spinning disk unit and the EMCCD camera. Target nuclei of embryos expressing NMY-2::GFP were irradiated with UV laser before the onset of curve formation, and fluorescence at the center plane of embryo was imaged with exposure time 150 ms. From the 2D images captured 8 min after the onset of the two-cell stage, the cortex of AB and P_1_ were manually traced using ImageJ, the mean pixel intensities were calculated, and background intensities were subtracted from them.

### Acquisition of cell shapes for computer simulation

GFP::PH embryos were stained for their eggshell with a 1 mg/ml solution of tetramethylrhodamine isothiocyanate-dextran (TRITC-dextran, Sigma T1037). The embryos were immediately rinsed with M9 buffer and mounted with poly-l-lysine. Two-color imaging of GFP and TRITC was performed with a Leica TCS SP2 AOBS confocal laser scanning microscope with a Leica ×63 NA1.20 water immersion objective lens. XY resolution was 0.244 µm/pixel. A single Z-stack consisted of 42–48 slices separated by 0.733 µm, and its acquisition took 20–24 seconds. In order to compensate for a weak fluorescence signal at the bottom of the embryos, we used higher laser power and detector sensitivity when imaging a deep part of the embryos. The confocal pinhole was set to 2.0 Airy units. Recording was performed 8 min after the completion of P_0_ cytokinesis.

For extracting contours of the plasma membrane, the confocal image stacks were smoothed by a Gaussian filter and scaled to cubic voxels of 0.733 µm. Segmentation of the image stack was performed using an in-house program for a 3D watershed algorithm [31]. Three voxels that represented AB, P_1_ and the exterior was manually selected and input into the program as markers. The program's output was segmentation of the image stack into the AB, P_1_, exterior regions and boundaries between them. All segmentation results were visually inspected and confirmed to have no serious errors. The eggshell contour was similarly extracted. The volume of a cell region, *V*, was computed as *V* = *N*_cell_+*N*_bnd_/2, where *N*_cell_ is the voxel number of the cell region, and *N*_bnd_ is the number of the boundary voxels surrounding the cell region. This formula had errors of less than 1% in a numerical study (data not shown).

### Computer simulation

Simulation was performed using Surface Evolver [32]. The two cells were modeled as two polyhedra that shared part of their surfaces, and they were confined to an ellipsoid using a one-sided constraint. The ellipsoid was defined by least squares fitting to the contour of the eggshell. The initial configuration was set to two cubes that shared one of their six faces. They were positioned in the center of the ellipsoid and aligned along the major axis. Each cube was 20 voxels (14.7 µm) on each side. The energy of each surface was defined as a product of its surface tension and surface area. The total energy was defined as a sum of the energy of the all surfaces. The total energy was minimized using gradient descent, and three rounds of triangle subdivision were performed in between. During the minimization, the volume of each polyhedron was constrained to the measured volume of the corresponding cell.

The final shape of the energy minimization was superimposed on the contour of the plasma membrane using the iterative closest point (ICP) algorithm [33]. ICP takes two point sets as input and returns translation, rotation and point-to-point correspondence that minimize the total distance between the corresponding points. Vertices of the polyhedra and voxels of the membrane contour were used as the input point sets. As a measure of shape coincidence, we computed root mean square error (RMSE) from the remaining distances between the corresponding points.

Because the unit of surface tension was arbitrary in our model, the parameter *γ*_P1_ was fixed to 1.0. For the homogeneous tension model, the other two parameters *γ*_AB_ and *γ*_cnt_ were also set to 1.0. For the heterogeneous tension model, various combinations of the parameters were tested for their ability to mimic target cell shape (Figure S1). The parameters *γ*_AB_ and *γ*_cnt_ were moved in the range from 0.2 to 10.0 in increments of 0.2. To prevent cells from detaching or engulfing each other, the surface tension parameters must satisfy the inequality |*γ*_AB_ − *γ*_P1_|≤*γ*_cnt_≤*γ*_AB_+*γ*_P1_. This requirement reduced the number of combinations to 514. For each combination, energy minimization was conducted, and the RMSE was computed. We selected the combination that gave the smallest RMSE.

### RNA interference

RNAi experiments were performed by injection of double-stranded RNA (dsRNA) to adult hermaphrodites. Sense and antisense RNA were transcribed in vitro using T3 and T7 RNA polymerases (Promega), mixed, and annealed. Templates for the transcription were PCR-amplified from genomic regions and purified using QIAGEN gel extraction kit. For primers, we used the same sequences as the study of Sönnichsen et al. [34]. Synthesized dsRNA were ethanol precipitated, dissolved in water, and filtrated by TAKARA SUPREC-01. For a mock, filtrated water was used. DsRNAs were injected into gonads bilaterally, and the injected worms were incubated at 22°C. After 24–30 hours from the injection, embryos were harvested, mounted using agar pad, and imaged with DIC optics at 5-s intervals. To define cell size symmetry, we measured cell size immediately after the completion of P_0_ cytokinesis because contact surface was flat at that time. Because the contact surface was flat, we assumed that cell size could be estimated by cell length along the AP axis. The cell lengths along the AP axis of AB and P_1_ were measured respectively from DIC images that were taken immediately after the completion of P_0_ cytokinesis using ImageJ. If the length difference between AB and P_1_ was within 10%, we defined that these cells had symmetric cell size. As the criterion for flat or curved contact surface, the angle formed by one endpoint, middle point, and the other endpoint of the contact was measured. If the angle was smaller than 160°, we defined that the contact surface was curved.

## Supporting Information

Figure S1 **A flow chart of parameter optimization.** Our procedure for finding the surface tension parameters that makes the model shape most similar to the target cell shape. The input is a stack of non-time-lapse 2D images of GFP::PH embryos. Red, the eggshell; green, the plasma membranes or their computationally extracted contours; orange, the legitimate region in the search space; magenta, model surfaces; dotted, an ellipsoid of least-squares fit to the eggshell contour.
(TIF)

Video S1 **Time-lapse movie of laser-induced cell fusion before curve formation.**
(MOV)

Video S2 **Time-lapse movie of laser-induced cell fusion after curve formation.**
(MOV)
