# Supplementary figures and images for: Cell-to-Cell Heterogeneity in Cortical Tension Specifies Curvature of Contact Surfaces in Caenorhabditis elegans Embryos
Source: PLoS One. 2012 Jan 10;7(1):e30224. doi: 10.1371/journal.pone.0030224 (PMC3254656; doi:10.1371/journal.pone.0030224)

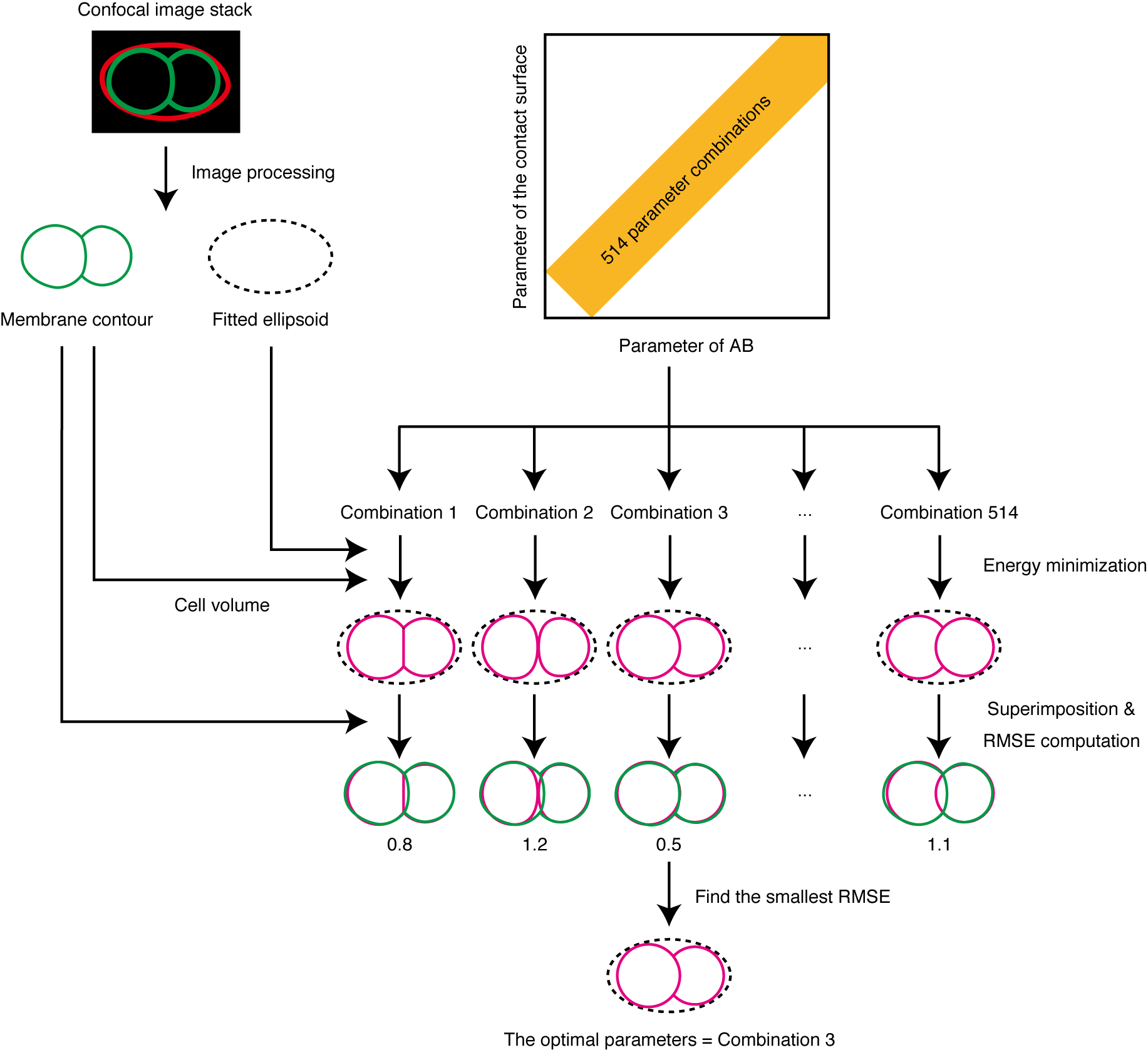

Supplement: Figure S1 — A flow chart of parameter optimization. Our procedure for finding the surface tension parameters that makes the model shape most similar to the target cell shape. The input is a stack of non-time-lapse 2D images of GFP::PH embryos. Red, the eggshell; green, the plasma membranes or their computationally extracted contours; orange, the legitimate region in the search space; magenta, model surfaces; dotted, an ellipsoid of least-squares fit to the eggshell contour. (TIF) [file pone.0030224.s001.tif]
